# Secondary Electrons in Gold Nanoparticle Clusters and Their Role in Therapeutic Ratio: The Outcome of a Monte Carlo Simulation Study

**DOI:** 10.3390/molecules27165290

**Published:** 2022-08-19

**Authors:** Hanan Akhdar, Reem Alanazi, Nadyah Alanazi, Abdullah Alodhayb

**Affiliations:** 1 Department of Physics, College of Science, Imam Mohammad Ibn Saud Islamic University, Riyadh 11623, Saudi Arabia; 2 Department of Biomedical Technology, College of Applied Medical Sciences, King Saud University, Riyadh 11433, Saudi Arabia; 3 Department of Physics and Astronomy, College of Science, King Saud University, Riyadh 11451, Saudi Arabia; 4 Research Chair for Tribology, Surface, and Interface Sciences, Department of Physics and Astronomy, College of Science, King Saud University, Riyadh 11451, Saudi Arabia

**Keywords:** gold nanoparticle, proton therapy, Geant4, secondary electrons

## Abstract

Gold nanoparticles (GNPs) are used in proton therapy radio-sensitizers to help increase the dose of radiation to targeted tumors by the emission of secondary electrons. Thus, this study aimed to investigate the link between secondary electron yields produced from a nanoshell of GNPs and dose absorption according to the distance from the center of the nanoparticles by using a Monte Carlo model. Microscopic evaluation was performed by modeling the interactions of secondary electrons in a phase-space file (PSF), where the number of emitted electrons was calculated within a spherical GNP of 15 nm along with the absorbed dose near it. Then, the Geant4-DNA physics list was used to facilitate the tracking of low-energy electrons down to an energy below 50 eV in water. The results show a remarkable change in the number of secondary electrons, which can be compared at concentrations less than and greater than 5 mg/mL, with increased secondary electron production exhibited around NPs within a distance of 10–100 nm from the surface of all nanospheres. It was found that there was a steep dose enhancement drop-off up to a factor of dose enhancement factor (DFE) ≤ 1 within a short distance of 100 nm from the surface of the GNPs, which revealed that the dose enhancement existed locally at nanometer distances from the GNPs. Overall, our results indicate that the physical interactions of protons with GNP clusters should not be considered as being directly responsible for the radio-sensitization effect, but should be regarded as playing a major role in NP properties and concentrations, which has a subsequent impact on local dose enhancement.

## 1. Introduction

Proton therapy is primarily aimed at increasing the therapeutic ratio by elevating the local dose to targeted tumors [1]. This is based on the considerable progress made in the past few years in the use of solid metal-based nanoparticles (NPs) as radio-sensitizers, resulting in the emission of photoelectrons and Auger electrons [2]. In particular, gold nanoparticles (GNPs) are known for their high cross-sectional area and good biocompatibility, and therefore have gained popularity in radiotherapy [3,4]. 

The efficiency of the treatment performed with the application of gold nanoparticles was proved by the results of studies by Liu et al. [5] and Tran et al. [6], who observed significant decreases in cell survival. Furthermore, earlier studies by Herold et al. [7] and Heinfeld et al. [8] found an increased biologically effective dose. Kim et al. [9], in an experimental study involving mice with adenocarcinoma, showed a one-year survival of over 50%. Besides experimental studies, a modeling investigation has been also reported. Lin et al. [10] developed a biological model aiming at studying the survival changes of irradiated cells due to the radio-sensitization effect of gold nanoparticles using proton beams. The study concluded that it is possible to better improve proton radiotherapy using GNPs if they can be absorbed into cells, especially the cell nucleus.

The increase in the total absorbed dose with the application of GNPs is attributed to the production of secondary electrons within them. In this regard, Leung et al. [11] reported an increase in secondary electron generation and irregular changes in the presence of GNPs in a water phantom. Likewise, Walzlein et al. [12] investigated the radial dose of a single GNP bead model in water, and they found that the radial dose decreased rapidly according to the distance from the NPs. The results of a simultaneous study by Lin et al. showed that the radiation dose had a value of 2–3 close to the NP, and it reached a plateau of approximately 14 at greater distances from the NP [13]. Jones et al. [14] used the EGSnrc code in the Monte Carlo simulation to quantify the dose distribution from secondary electron dose point kernels within a GNP-loaded tumor. They demonstrated that the microscopic dose around a GNP was enhanced up to a factor of more than 100.

Carter et al. [15] studied the importance of the localization of NPs and how this affects the observed radio-sensitization. They concluded that a high concentration of electrons and radicals is created around the NPs, and in order to achieve radio-sensitization, either these nanomaterials should be delivered precisely to specific sites, or their concentration should be increased.

The simulation in our study was based on a Geant4 Monte Carlo code, which was adopted following previous studies [16,17]. The simulation was used to describe the physical reaction of protons with GNPs, which depends on the region of the defined geometry, in an energy range that allows for the tracking of protons, electrons, photons, etc. Here, the Livermore low-energy physics list was used to track the protons, whereas the interactions of secondary electrons leaving the GNPs within the surrounding water were modeled using the Geant4-DNA list [18]. Thus, this work explains that the increased dose is due to GNPs being in a random distribution, while it also illustrates the effect of GNP concentration as clusters on the dose deposition and secondary electron yield [16,19]. The evaluation of secondary electrons emitted by GNPs remains the important concern, as it can help to determine the effective dose for certain diseases. Therefore, the purpose of the study was to estimate the role of GNPs in the amount of the absorbed dose according to the distance from the center of the nanoparticles using a Monte Carlo model.

## 2. Materials and Methods

### Simulation Setup

**Figure 1:**
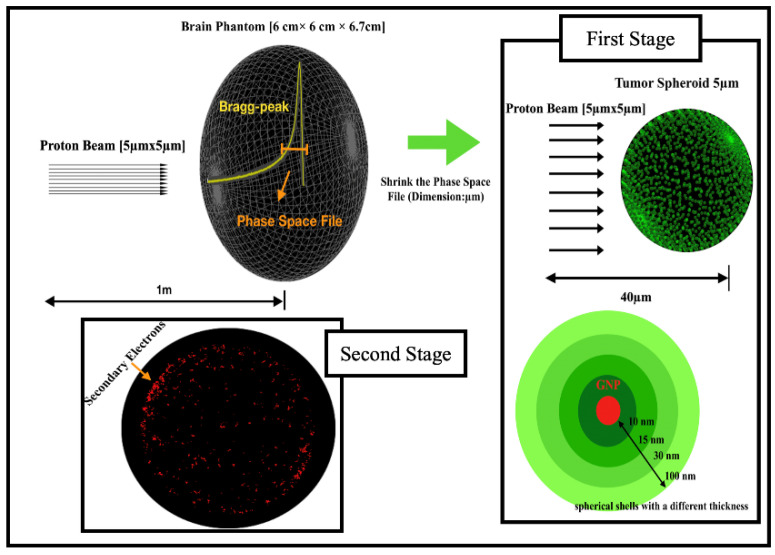
Schematic diagram of geometry used in this study. First stage: recorded phase-space files (PSFs) were rescaled to GNP size to expose them and considered as new sources to investigate dose deposition around GNPs in spherical shells with a thickness of 100 nm, in addition to counting secondary electrons recorded in spherical shells. Second stage: studying the spectrum of electrons in water.

**Figure 2:**
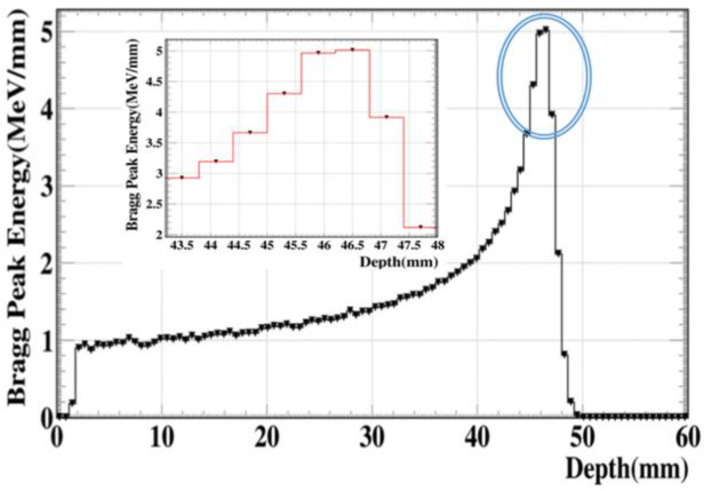
Physical energy deposition profile in Bragg peak region for mono-energetic 100 MeV proton beam at 60 mm depth.

**Figure 3:**
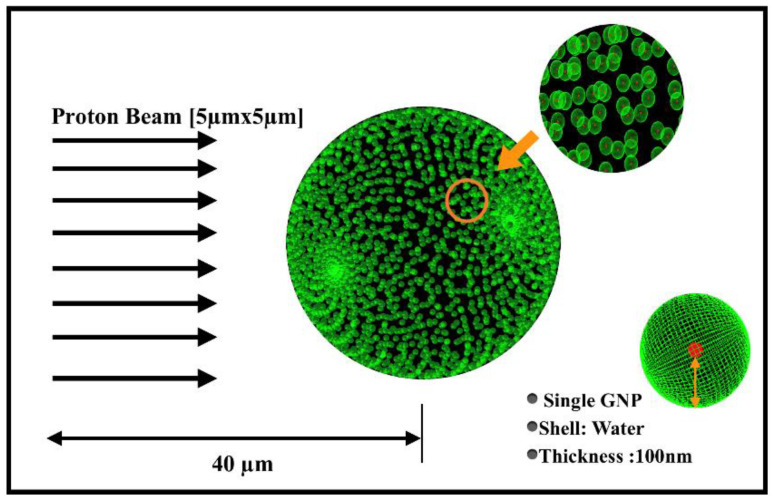
Visualization of 5 µm spherical tumor enveloped by randomly distributed GNP clusters, with 40 nm distance between surface source and central axis of tumor.

A hundred thousand protons with an energy of 100 MeV in a 2D shape (5 µm × 5 µm) were released and placed 1 m away from a water brain phantom with dimensions of 6 cm × 6 cm × 6.7 cm [20,21], and the whole apparatus was positioned within an air-filled volume with dimensions of 120 cm × 120 cm × 120 cm (Figure 1). Accordingly, the energy deposition profiles of the proton beams were calculated at a brain depth at 60 mm. We found that the Bragg peak region covering the tumor region was located at a depth of about 47 mm, which was in the target region between 45.5 and 47 mm; 4 MeV at 45.5 mm to 5 MeV at 47 mm. The energy deposition profile of the proton beam is shown in Figure 2 [22]. The phase-space file (PSF) was recorded at the Bragg peak region that contained the kinetic information of the transiting protons. The phase-space sources were shrunk from millimeter (mm) to micron (µm) scale, as represented in Figure 1, by reducing the X- and Y-coordinates of the traversing protons by a factor of 10^3^ for each depth. A 5 µm microsource of protons as a shrunken phase-space source of traversing protons in the microscopic study was acquired while the kinetic information of the broad proton beams traveling in water was retained. The microsource was paralleled to cover a water sphere with a diameter of 5 µm to represent the tumor region [23]. In the two subsequent microscopic stages as in Figure 3, the effect of the tumor-encapsulated nanoparticles and the resulting electrons emitted from the surface of GNPs in the spherical shell were studied and counted, and then the spectrum of electrons in the water was investigated.

In this microscopically performed study, the geometry of the simulation case was based on previous studies to calculate the dose absorption around uncoated spherical GNPs with a diameter of 15 nm, which is an optimal GNP size to maximize enhanced irradiation with a 5 MeV proton beam [13,17,24]. The study consisted of two stages: In the first stage, the secondary electrons emitted from each GNP were recorded in a phase-space file (PSF), while Livermore low-energy physics models were used to track the interactions between protons and GNPs in order to calculate the dose around the NPs. The dose deposited in the water by the secondary electrons was recorded along with the number of electrons on each GNP in the clusters. Following this, the output data were inputted from the first stage of the simulation to model the physical interactions along with the secondary electrons escaping the NP surfaces to the surrounding water. In the second stage, the Geant4-DNA physics list was used, which enabled the tracking of low-energy electrons down to an energy below 50 eV. At this stage, all the cluster components were modeled as consisting of water, and this was chosen as if it were the only medium available for the full Geant4-DNA physics and chemistry models [17], whereas the proton beam was modeled with a box shape of dimensions of 5 μm × 5 μm, located 40 μm from the target region in the microscopic first stage.

## 3. Results

**Figure 4:**
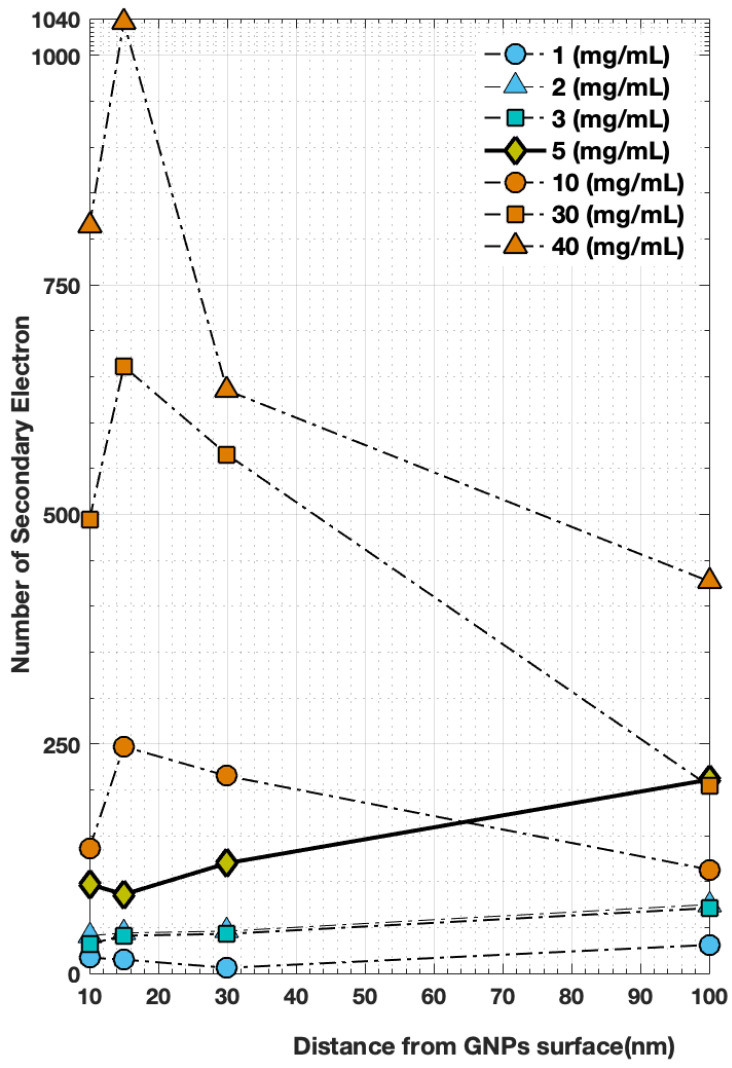
Calculated number of secondary electrons on GNP surface at cluster concentrations of 1, 2, 3, 5, 10, 30, and 40 mg/mL as a function of radial distance.

**Figure 5:**
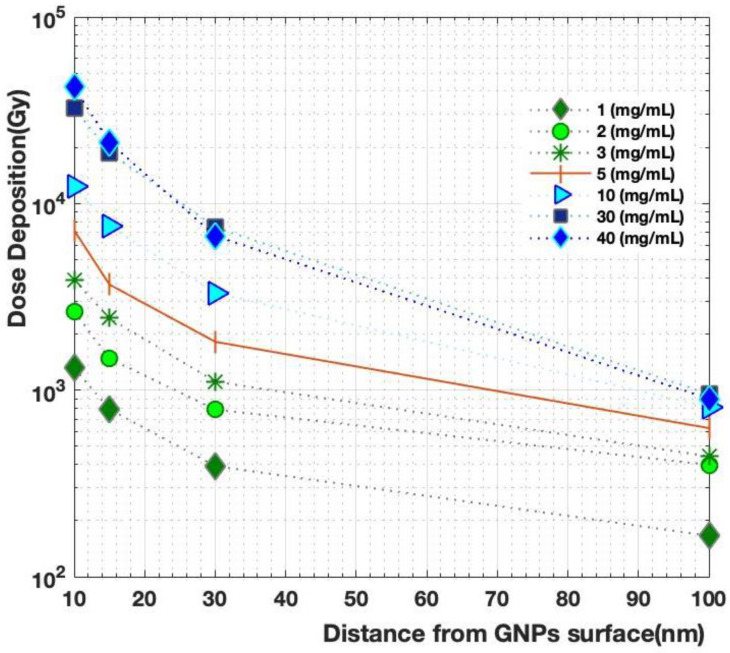
Simulation of the emission of electrons from GNPs with different radii (10, 15, 30, and 100 nm) consisting of water with several GNP cluster concentrations.

The electrons escaping from GNP clusters were simulated according to GNP concentrations of 1, 2, 3, 5, 10, 30, and 40 mg/mL. The scoring volume was represented by the shell, and the effective energy deposition of each NP within the GNP clusters was obtained. We observed a significant change in the number of secondary electrons in the nanometer range according to the distance from the surface of each NP, as shown in Table 1. Figure 4 presents the difference in electrons counted according to the cluster concentration based on the distance from the cluster surface, with the generation of electrons exhibiting a clear difference depending on the distance at different concentrations. Figure 5 shows the dose deposition as a function of cluster concentration from the surface of each NP. We observed that when the concentration was <5 mg/mL, the greater the distance, the greater the generation of secondary electrons. In the case of concentrations of >5 mg/mL, the generation of secondary electrons decreased sharply with increased distance, especially when the concentration was >30 mg/mL. The electron yield ratio began to exhibit marked differences when the bulk aggregation concentration reached the peak of 1000 for the GNP cluster distribution at 40 mg/mL, and the observed decrease was due to the merging of clusters at a distance beyond 100 nm, where the total number of groups decreased to <100.

In the case of concentrations of 1, 2, and 3 mg/mL, at a distance of 10 nm from the surface, the numbers of secondary electrons generated were approximately 17, 41, and 32, whereas at a distance of 15 nm, there were 15, 44, and 41; at 30 nm, there were 6, 46, and 43; and at 100 nm, there were 31, 75, and 71. A difference appeared at concentrations of 10, 30, and 40 mg/mL at a distance of 10 nm from the surface, with approximately 136, 494, and 814 secondary electrons generated, respectively, whereas at a distance of 15 nm, there were 247, 661, and 1036; at 30 nm, there were 215, 565, and 635; and at 100 nm, there were 113, 204, and 427, respectively. The largest change in electron generation appeared at concentrations of 10, 30, and 40 mg/mL; more significant changes were detected at the higher concentration, whereas they were relatively weaker compared with the lower concentrations, as shown in Figure 4.

Additional spherical water volume surrounding GNPs with different radii (10, 15, 30, and 100 nm) was added to determine the absorbed radiation dose in gray (Gy). This geometry was particularly useful for isolating the effect of electrons generated from the GNPs without any influence from the surroundings and comparing them to the reference material, which in this case was water. In other words, proton interactions could only take place in the gold or water if we wanted to record the energy deposition caused by secondary electrons in this particular region. To achieve this, we counted the secondary electrons emitted from each GNP and then compared the results with the previously published values. As the distance from the GNP surface increased, the absorbed dose decreased sharply. Figure 5 shows dose deposition as a function of cluster concentration from the surface of each NP. Here, the dose was reduced from 7194 to 626 at a concentration of 5 mg/mL, from 1313 to 165 at a concentration of 1 mg/mL, from 1642 to 307 at a concentration of 2 mg/mL, and from 3905 to 441 at a concentration of 3 mg/mL.

**Figure 6:**
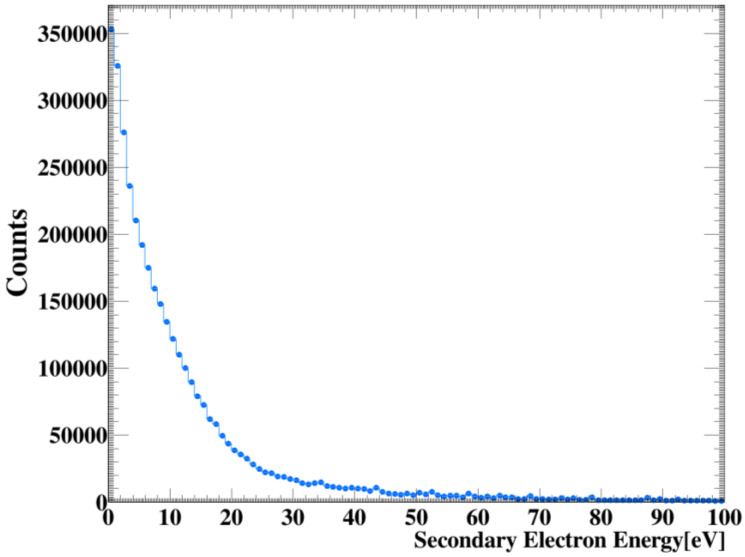
Electron emission spectrum on gold surface as a function of GNP clusters at a concentration of 5 mg/mL.

It was important to examine and record the electron emission spectrum of the GNP clusters at a concentration of 5 mg/mL within a range of 100 nm from the surface, as we wanted to consider the contributions of the scattered electrons inside the phantom, count them, and take into account the dose deposition of these electrons in the water medium. Here, we observed the contributions of the electrons in the maximum range of ~1–10 eV when traced in water, as shown in Figure 6, given that they were mainly responsible for the biological effects, and considering that they were hypothesized to be mainly responsible for those effects that did not exceed a range of a few nanometers from their position.

## 4. Discussion

This study aimed to estimate the effect of GNPs on increased dosing by clustering GNPs with different concentrations and evaluating the number of secondary electrons they emitted by calculating the dose around them as recorded according to the envelope “shell” of each GNP and the surrounding water. This was conducted by applying a Monte Carlo simulation technique that involved the microscopic stage. 

In the study, the geometry of GNPs in clusters was randomly modeled in the tumor, since this is an intracellular distribution commonly observed in experiments [25,26]. When ascertaining the nanosensitization effect of the GNP clusters on the tumor under actual conditions (the geometric construction shown in Figure 3), it was found that the clustering of GNPs leads to high local dose concentrations.

The study was extended to calculate the number of secondary electrons emitted from the GNPs, which was calculated at each cluster concentration. The secondary electron count was low, with energy values of 2 keV or less, which dropped rapidly with increased distance from the GNP surface (distances > 100 nm), likely because protons have a significant radio-sensitizing effect close to GNPs, which in turn could increase the therapeutic ratio by delivering a large dose to the tumor.

The relationship between counting electrons and measuring the deposited dose based on the distance from the surface of GNPs at each concentration of clusters was determined, as shown in Table 1. The generation of electrons exhibited high linearity with increasing cluster concentration and decreasing distance from GNP surfaces at different concentrations. Meanwhile, as shown in Figure 4, the generation of additional electrons at short distances in the nanometer range led to an increase in the absorbed dose (Figure 6), which could increase the therapeutic ratio by delivering a large dose to the tumor region. As such, the GNP clusters used should be designed to accumulate as much as possible at the target to maximize the radio-sensitization effect. In this study, the effect of extra aqueous electrons around GNP clusters at the chemical stage and the estimation of resulting biological damage were not simulated. In fact, the research was limited to investigating the physical interactions among local dose deposits resulting from GNPs. The resulting models could be implemented in specific microscopic tumor models used in multiple therapeutic approaches for the effective enhanced treatment of targeted tumors.

## 5. Conclusions

The results of this study prove that the GNP concentration is directly proportional to the number of emitted secondary electrons, and the generation of additional electrons at short distances causes an increase in the absorbed dose. Therefore, the therapeutic dose can potentially be increased, which is useful in the treatment of diseases. However, more evidence should be provided regarding the effect of GNP concentration on biological tissues, so that the impact of secondary electrons can be evaluated in terms of the detected effective dose, which will be an important issue for future research. This investigation, which expands on the link found between physical parameters and the effective dose, can be a major contribution to the clinical standards of radiotherapy for various types of tumors.

## Figures and Tables

**Table 1:** Number of secondary electrons at different GNP concentrations.

Geometry Configuration (Number of Gold Nanoparticles)	Distance from GNP Surface (nm)	Number of $e^{-}$ per Proton	Dose Deposition (Gy)
1918	10	17	1313
	15	15	791
	30	6	391
	100	31	165
3832	10	41	2642
	15	44	1477
	30	46	786
	100	75	307
5752	10	32	3905
	15	41	2429
	30	43	1110
	100	71	441
9588	10	97	7194
	15	86	3682
	30	126	1816
	100	211	626
19,172	10	136	12,412
	15	247	7562
	30	215	3321
	100	113	812
57,518	10	494	32,581
	15	661	18,804
	30	565	7477
	100	204	952
76,692	10	814	42,174
	15	1036	21,317
	30	635	6706
	100	427	898

## Data Availability

The data that support the findings of this study are available from the corresponding author upon reasonable request.
